# Detection of Adverse Medicine Events by Pharmacists in Residential Aged Care Facilities: Secondary Analysis of Data From ReMInDAR Trial

**DOI:** 10.1002/pds.70261

**Published:** 2025-11-06

**Authors:** Abebe Basazn Mekuria, Renly Lim, Andre Q. Andrade, Debra Rowett, Elizabeth E. Roughead

**Affiliations:** ^1^ Quality Use of Medicines and Pharmacy Research Centre, UniSA Clinical and Health Sciences University of South Australia Adelaide Australia

**Keywords:** adverse medicine events, medication review, medication safety, older people, patient reported outcomes, pharmacy services

## Abstract

**Background:**

Pharmacists often identify symptoms during medication reviews that may or may not be adverse medicine events (AMEs), but these have not yet been quantified. This study aimed to quantify the extent of these symptoms representing AMEs by comparing them with a known set of AMEs and symptoms listed in existing medicine‐related symptom assessment tools.

**Method:**

A secondary analysis of data from the Reducing Medicine‐Induced Deterioration and Adverse Reactions (ReMInDAR) trial was conducted. Adverse events or symptoms were extracted from pharmacists' progress notes, and their frequency and Medicine Likeliness Ratio (probability of being medicine‐related) were determined. Pharmacist‐recorded adverse events were compared to a subset of AMEs identified by a clinical panel, and agreement was assessed using Cohen's *κ*. Pharmacist‐recorded adverse events or symptoms were also compared with those in the PHArmacotherapeutical Symptom Evaluation‐20 questions (PHASE‐20), and the Patient Reported Outcome Measure, Inquiry into Side Effects (PROMISE).

**Result:**

Pharmacists recorded 3.1 symptoms per person; 68.8% of the symptoms had a medicine‐likeness ratio ≥ 40.0%. The most prevalent medicine‐related events recorded by pharmacists included falls (13.6%), swelling (7.1%), constipation (5.4%), nocturia (4.2%), shortness of breath (4.0%), bleeding (4.0%), nausea and vomiting (3.1%), dizziness (2.8%), drowsiness (2.3%), and rash (2.0%). Of the subset of 273 AMEs identified by the panel, 14.7% corresponded to adverse events recorded by pharmacists. The agreement between pharmacist‐recorded and panel‐identified AMEs was significant but low (*κ* = 0.074, *p* = 0.008). The majority of frequently detected medicine‐related symptoms were in PROMISE (56.4% of recorded AMEs) and PHASE‐20 (81.3% of recorded AMEs).

**Conclusion:**

While pharmacists recorded a notable number and variety of adverse events or symptoms, underreporting and discrepancies were still observed. As items in the PHASE‐20 aligned with most recorded events, further research is warranted to determine if it can help pharmacists in improving the detection and monitoring of AMEs.

**Plain Language Summary:**

Pharmacists often notice symptoms during medication reviews that may or may not be caused by the medicines. This study aimed to measure how often the symptoms reported by pharmacists were side effects of the medicines in use. We looked at data from a previous trial in aged care homes and reviewed the notes pharmacists wrote about possible symptoms or side effects. We then compared these to a list of known medicine‐related problems identified by medical experts. On average, pharmacists recorded about three symptoms per person, and more than two‐thirds of these were likely to be related to medicines. The most common side effects or symptoms included falling, swelling, difficulty passing stools, needing to urinate at night, feeling short of breath, bleeding, feeling nausea or vomiting, feeling dizzy, feeling sleepy, and skin rashes. However, only a small number of the problems recorded by pharmacists matched the expert‐reviewed list. Most of the symptoms reported by pharmacists were also included in PHASE‐20 symptom checklists, which is designed to help identify medicine‐related symptoms. This suggests that this tool might support pharmacists to detect and track medicine‐related problems. Further research is needed to explore how best to use this tool in practice.

**Summary**

- Pharmacists in aged care facilities recorded an average of 3.1 adverse events or symptoms per resident over the year, of which 68.8% were deemed likely to be AMEs.
- Underreporting of AMEs by pharmacists was evident, with 14.7% of panel‐identified AMEs recorded in pharmacists' notes.
- There was a low but statistically significant agreement between pharmacist‐documented AMEs and those identified by a clinical panel (*κ* = 0.074, *p* = 0.008).
- The majority of frequently detected medicine‐related events recorded by pharmacists were in current available medicine‐related symptom assessment tools.

## 1 Background

Older adults residing in aged‐care facilities are vulnerable to adverse medicine events (AMEs) due to age‐related changes in physiology, pharmacodynamics, and pharmacokinetics, as well as the presence of comorbidity and polypharmacy [1, 2, 3, 4]. Residents in aged care facilities take more medicines compared to younger individuals and the elderly living in the community who have similar health problems [5], which may increase the occurrence of medicine‐related adverse events (AEs) [6, 7]. AMEs refer to any negative impact or harm caused by medication errors or adverse drug reactions (ADRs) associated with the use of medicines [8]. AMEs increase patient morbidity, mortality, and length of stay in the hospital, decrease quality of life, increase health‐related costs, and affect treatment outcomes [9, 10, 11]. The detection and resolution of AMEs among patients require the expertise of pharmacists and collaboration within a multidisciplinary team [12, 13]. A systematic review examining factors influencing medication safety revealed that lack of access to pharmacists and insufficient interdisciplinary collaboration had a negative impact on medication safety, including prevention of AMEs [14].

Pharmacists can play a role in reducing medicine‐related harms or AMEs in the older population [15, 16, 17]. Frequent visits by pharmacists contributed to the low incidence of AMEs in nursing homes [18, 19]. A study conducted in the USA in 2018 found that the accessibility of consultant pharmacists outside of visit days had a significant negative association (OR = 0.73, CI: 0.63–0.86) with AME incidence [18]. Another study conducted in Australian aged care facilities found that the proportion of residents reporting new medicine‐related problems or symptoms declined during repeated pharmacist sessions (from 59% at Visit 2 to 28% at Visit 6, *p* < 0.01) [19]. Given their potential to detect and prevent medicine‐related problems including AMEs, pharmacists' identification of patient‐reported signs and symptoms may be advantageous for early detection and resolution of AMEs experienced by patients [20, 21]. Proactive early recognition and amelioration of medicine‐related signs and symptoms can prevent more serious medicine‐related harm among patients [22]. There are currently various types of AMEs detection methods available, including chart review, clinical judgment, trigger tools, and self‐reporting tools [23, 24, 25]. Self‐reporting medicine‐related symptom assessment tools, which encompass a range of medicine‐related symptoms, have been developed to assist health professionals including pharmacists in detecting medicine‐related events [26, 27].

Pharmacists often identify symptoms during medication reviews, which may or may not be AMEs [26, 27, 28]. As of the date of this study, no studies have assessed the extent to which the symptoms recorded by pharmacists represent AMEs. Therefore, this study aimed to quantify the extent of medicine‐related symptoms recorded by pharmacists by comparing them with a known set of AMEs (gold standard) and the symptoms listed in medicine‐related symptom assessment tools. The result of this study will inform to prioritize specific types of symptoms or AMEs for monitoring during pharmacist services, as well as the selectin of a tool that is aligned with routine practice and comprehensive enough to capture the range of AMEs recorded by pharmacists.

## 2 Methods

### 2.1 Study Design and Setting

This study was a secondary analysis of data from the Reducing Medicine‐Induced Deterioration and Adverse Reactions (ReMInDAR) trial, which aimed to evaluate the effectiveness of a pharmacist‐led intervention in reducing medicine‐induced deterioration and adverse reactions [29]. The ReMInDAR trial was an open‐label, multicenter randomized controlled trial involving 248 residents from 39 aged care facilities [29]. Residents in the control group received usual care, while those in the intervention group received pharmacist‐led services [30]. The trial details have been fully described in the trial protocol and in a published study reporting the primary outcomes [29, 30]. Ethics approval was obtained prior to participant recruitment from the Human Research Ethics Committee of the University of South Australia (ID: 0000036440) and the University of Tasmania (ID: H0017022) [30].

In this secondary study, all residents who received pharmacist‐led services in the trial were included (*n* = 115). The quantification of medicine‐related symptoms or AMEs recorded by pharmacists was done by comparing the symptoms identified by pharmacists with a known set of AMEs (gold standard) and symptoms listed in existing medicine‐related symptom assessment tools.

### 2.2 Pharmacist Recorded AMEs

In the ReMInDAR trial, trained pharmacists provided services every 8 weeks over 12 months for enrolled residents. During the trial, a total of 575 individual pharmacist–resident sessions were conducted, and 309 recommendations were provided to either change or monitor medicine use [29]. During each visit, pharmacists reviewed residents' care records to identify any new illness, AEs, or any signs or symptoms noted in the care record since the last assessment. Additionally, the pharmacists reviewed the medication chart to identify any changes in medication use. The pharmacist met with the resident and care staff to discuss concerns about the residents' health and medications, and assessed changes in 24‐h movement behavior, handgrip strength, and cognition using validated instruments [29, 30]. If medicine‐induced deterioration was considered clinically significant and medication‐related problems were identified, the pharmacists liaised with the participants' doctors to discuss the residents' health status and provide recommendations. The intervention pharmacists documented all services provided, findings observed, and recommendations made in their progress notes following each service session with residents.

During the trial, the intervention pharmacists were asked to record in their progress notes whether the AEs or symptoms they identified were related to medicine use among residents. The intervention pharmacists indicated “yes,” “unsure,” or “no” for each recorded AE/symptom. In this study, a research pharmacist reviewed all progress notes recorded during the trial and extracted information on AEs or symptoms, their corresponding dates, and the pharmacists' assessments of medicine‐relatedness using a preformatted data extraction form. The extracted data were then reviewed by a second pharmacist, who compared them with the original notes, and any discrepancies were resolved through discussion.

### 2.3 Gold Standard AMEs

Research assistants identified AEs, which were then adjudicated as medication‐related events by a clinical panel considered to be the gold standard AMEs. The trained research assistants reviewed and recorded the AEs from the residents' care records using keyword search terms, including falls (both non‐injurious and injurious, including fractures), bleeding, bruising, confusion, dizziness, delirium, and fecal impaction [31]. Two pharmacists, both with expertise in clinical pharmacy and medication safety for older people, independently screened all AEs recorded by the research assistants to produce a shortlist of potential AEs related to medicine and coded the causality of these shortlisted AEs using the Naranjo Probability Scale criteria [32]. Discrepancies between the clinical pharmacists in causality assessment were resolved by a third pharmacist. AEs that were considered by research pharmacists to be possible, probable, or definite medicine‐related events were then subject to clinical panel review. An expert clinical panel, consisting of two pharmacists and a doctor, further reviewed each potential medicine‐related AE identified by the clinical pharmacists to determine the likelihood of it being medicine‐related [29]. The clinical panel then rated the AMEs as definite, probable, possible, or doubtful using the Naranjo Probability Scale criteria [32]. AEs in the intervention group that were rated as definite, probable, or possible by the clinical panel were considered medicine‐related and served as the gold standard AMEs for comparison with those recorded by the intervention pharmacists during the trial. There were 273 gold standard AMEs identified by the panel during the trial, including 139 falls events, 56 bleeding events, 29 bruising events, 24 dizziness events, 10 confusion events, 5 constipation events, 3 rash events, 3 cough events, 2 nocturia events, and 1 nausea/vomiting event.

### 2.4 Data Analysis

#### 2.4.1 Type of Adverse Events or Symptoms Detected by Pharmacists

The frequency of each symptom recorded by pharmacists was calculated. In addition, the Medicine Likeliness Ratio, which represents the probability of symptoms being medicine‐related, was calculated by dividing the total number of each type of symptom categorized as “yes” and “unsure” by the total number of symptoms recorded by pharmacists. In our study, the cutoff points for the most prevalent medicine‐related symptoms frequency were ≥ 1.0% and a medicine‐likeliness ratio of ≥ 40.0% [26, 27], thereby being considered a medicine‐related event.

#### 2.4.2 Proportion of AMEs Detected by Pharmacists Compared to the Gold Standard

For the subset of AMEs for which we had a gold standard, each AE or potential medicine‐related symptom extracted from the pharmacist notes was matched, where possible, with the corresponding gold standard AMEs using individual study IDs and dates spanning a two‐month window either side of the date of the event identified by the clinical panel. The proportion of AMEs recorded by the pharmacist was determined.

We assessed the agreement between pharmacist records and panel‐identified AMEs using Cohen's *κ*, which measures the level of agreement beyond chance. The presence or absence of an AME recorded by the pharmacist in the notes was compared with the gold standard, and *p* < 0.05 were considered statistically significant [33]. *κ* value was interpreted as follows: < 0.20 = slight agreement, 0.21–0.40 = fair agreement, 0.41–0.60 = moderate agreement, 0.61–0.80 = substantial agreement, and > 0.80 = almost perfect agreement [34].

#### 2.4.3 Compare Frequently Detected Symptoms With Items Listed in Medicine‐Related Symptom Rating Tools

A descriptive comparison was conducted between the most frequently detected medicine‐related symptoms that were recorded by pharmacists and symptoms listed in the medicine‐related symptom tools, the Patient Reported Outcome Measure, Inquiry into Side Effects (PROMISE) [26] and the PHArmacotherapeutical Symptom Evaluation, 20 questions (PHASE‐20) [27]. Evidence from literature review, the PROMISE and PHASE‐20 tools have been validated and are designed to assess potential medicine‐related symptoms in older adults [26, 27].

## 3 Results

### 3.1 Types of Adverse Events or Symptoms Detected by Pharmacists

Table 1 provides a list of side effects and AEs recorded by pharmacists. Pharmacists recorded a total of 353 potential medicine‐related symptoms among 115 residents, averaging 3.1 per resident. The medicine‐likeness ratio tells us the pharmacists' assessment of definite or possible side effects. Out of the total symptoms recorded by pharmacists, 68.8% (243 out of 353) had a medicine‐likeness ratio of ≥ 40.0%, and 83.5% (203 out of 243) of them had a frequency of ≥ 1.0%, thereby being considered AMEs (Table 1).

**TABLE 1:** Type and frequency of adverse events or symptoms recorded by pharmacists in aged care facilities.

Type of adverse events/symptoms recorded by pharmacists	Frequency *n* (%) (*N* = 353)	Medicine likeliness ratio (%)
Pain^a^	51 (14.4)	13.7
**Fall**	**48 (13.6)**	**43.8**
**Swelling** ^b^	**25 (7.1)**	**64**
**Constipation**	**19 (5.4)**	**73.7**
**Nocturia**	**15 (4.2)**	**53.3**
**Bleeding**	**14 (4.0)**	**50**
**Shortness of breath**	**14 (4.0)**	**64**
**Nausea/vomiting**	**11 (3.1)**	**54.5**
**Dizziness**	**10 (2.8)**	**90**
**Drowsiness**	**8 (2.3)**	**87.5**
**Rash**	**7 (2.0)**	**57.1**
**Bruising**	**6 (1.7)**	**50**
**Diarrhoea**	**6 (1.7)**	**83**
**Cognitive impairment**	**6 (1.7)**	**50**
**Tiredness**	**6 (1.7)**	**50**
Depression	6 (1.7)	33
Dry eyes	6 (1.7)	33
Anxiety	5 (1.4)	20
**Confusion**	**4 (1.1)**	**75**
**Dry mouth**	**4 (1.1)**	**100**
Allergy	4 (1.1)	25
Burning sensation	4 (1.1)	25
Insomnia	4 (1.1)	0
Cough	3 (0.8)	33.3
Thrush	3 (0.8)	100
Anaemia	3 (0.8)	67
Itching	3 (0.8)	67
Headaches	3 (0.8)	33
Increase BP	3 (0.8)	33
Weight gain	3 (0.8)	33
Weight loss	3 (0.8)	33
High blood glucose	3 (0.8)	0
Visual impairment	3 (0.8)	0
Dermatitis	2 (0.6)	100
Neuropathy	2 (0.6)	100
Unconsciousness	2 (0.6)	100
Unsteady	2 (0.6)	100
Cataract	2 (0.6)	50
Cellulitis	2 (0.6)	50
Leg weakness	2 (0.6)	50
Loss of balance	2 (0.6)	50
Renal impairment	2 (0.6)	50
Sore throat	2 (0.6)	50
Variable INR	2 (0.6)	50
Vertigo	2 (0.6)	50
Difficulty swallowing	2 (0.6)	0
Abdominal discomfort	1 (0.3)	100
Eye irritation	1 (0.3)	100
Hallucination	1 (0.3)	100
Impotence	1 (0.3)	100
Low sodium level	1 (0.3)	100
Poor appetite	1 (0.3)	100
Potassium too low	1 (0.3)	100
Aneurysm	1 (0.3)	0
Eye pigmentation	1 (0.3)	0
Hypothyroidism	1 (0.3)	0
Low TSH	1 (0.3)	0
Restless legs	1 (0.3)	0
Seizures	1 (0.3)	0
Watery/itchy eyes	1 (0.3)	0

*Note:* Bolded adverse events or symptoms are considered the most prevalent medicine‐related symptoms (frequency ≥ 1.0% and medicine‐likeliness ratio of ≥ 40.0%).

Abbreviation: TSH = thyroid‐stimulating hormone.

^a^ It includes chest pain, abdominal pain, abdominal cramps, muscle pain, and back pain.

^b^ It includes swelling of the ankles, legs, and arms.

The most frequently recorded potential medicine‐related symptoms or events, with medicine‐likeness ratios above 40%, include falls (13.6%), swelling (7.1%), constipation (5.4%), nocturia (4.2%), shortness of breath (4.0%), bleeding (4.0%), nausea/vomiting (3.1%), dizziness (2.8%), drowsiness (2.3%), rash (2.0%), cognitive impairment (1.7%), tiredness (1.7%), bruising (1.7%), diarrhoea (1.7%), confusion (1.1%), and dry mouth (1.1%) (Table 1).

### 3.2 Proportion of AMEs Detected by Pharmacists Compared to the Gold Standard

The results in Table 2 highlight both underreporting and discrepancies between AMEs recorded by pharmacists and those identified by the clinical panel, the latter which served as the gold standard. Among the gold standard AMEs, 14.7% (40 out of 273 AMEs) corresponded to the AEs recorded by pharmacists. Out of 139 medicine‐related falls, 15.1% were recorded by pharmacists. There were some AEs recorded by the pharmacist that were not recorded by the panel (Table 2).

**TABLE 2:** Proportion and discrepancies of AMEs recorded by pharmacists compared to gold standard (panel‐identified) AMEs.

Type of AMEs	Gold standard AMEs^a^	Proportion of gold standard AMEs recorded by pharmacists,^b^ *n* (%)	Discrepancies between pharmacist‐recorded and panel‐identified AMEs		
			Recording date mismatch^c^	Not deemed AMEs by the panel^d^	Only pharmacist‐recorded AEs^e^
Fall	139	21 (15.1)	10	17	0
Bleeding	56	5 (8.9)	1	4	4
Bruising	29	3 (10.3)	0	1	2
Dizziness	24	5 (20.8)	0	2	3
Confusion	10	3 (30.0)	0	0	1
Constipation	5	2 (40.0)	2	1	14
Rash	3	0 (0.0)	1	1	5
Cough	3	0 (0.0)	0	2	1
Nocturia	2	0 (0.0)	0	1	14
Nausea/vomiting	1	1 (100.0)	0	5	5
Total	273	40 (14.7)	14	34	49

Abbreviations: AEs = adverse events; AMEs = adverse medicine events.

^a^ Adverse vents reviewed and deemed as medicine‐related by clinical panel.

^b^ Number and proportion of gold standard AMEs recorded by pharmacists.

^c^ Adverse events recorded by both pharmacists and the panel as AMEs but with different recorded dates (we examined the recording dates of AMEs recorded by pharmacists by comparing the date of recording of gold standard AMEs falling within a span of 2 months).

^d^ Adverse events recorded by pharmacists that were not classified as AMEs by the panel.

^e^ Adverse events recorded by pharmacists but not identified by the panel at all.

Cohen's *κ* statistic indicated a slight but statistically significant agreement between pharmacist‐recorded AMEs and clinical panel‐identified AMEs (*κ* = 0.074, *p* = 0.008).

### 3.3 Comparison Between the Most Frequently Detected Medicine‐Related Symptoms and Items Listed in the Medicine‐Related Symptom Tools

We conducted a qualitative comparison between the commonly recorded medicine‐related symptoms by pharmacists and those included in validated symptom assessment tools (PROMISE and PHASE‐20). The majority of common medicine‐related symptoms recorded by pharmacists were in the PROMISE (56.4%) and PHASE‐20 (81.3%) medicine‐related symptom assessment tools. The detailed comparison is provided in Data S1.

## 4 Discussion

Our study found that pharmacists recorded an average of 3.1 symptoms per resident, of which the majority (68.8%) were medicine‐related, as the medicine likeliness ratio was greater than 40%. This finding highlights that pharmacists can identify a notable number of potential medicine‐related symptoms in aged care facilities. However, there were some discrepancies and under‐detection of AMEs by pharmacists highlighting the need for strategies to improve the comprehensive identification and monitoring of AMEs during pharmacists' services.

In our study, the majority of the frequent medicine‐related symptoms recorded by pharmacists were among the most common and preventable AMEs in Australian aged care facilities [35]. Early identification, monitoring and resolution of medicine‐related symptoms by pharmacists may help to reduce further medicine‐induced deterioration. For example, early identification of dizziness or drowsiness may reduce the risk of falls, which was the most common AME recorded by pharmacists in our study and the most prevalent preventable AME among residents in aged care facilities [35, 36]. In turn, this may contribute to preventing fall‐related harm, including fractures, hospitalization, and death, as falls are known risk factors for these adverse health outcomes in older people [37, 38]. For the prevention of medicine‐induced harm, pharmacists will need to not only review patients' medications but also recognize and monitor signs and symptoms reported by patients. The symptoms reported by the patient can be indicative of AMEs [32, 39]. In our study, the majority of symptoms recorded by pharmacists exhibited a high likelihood ratio of being medicine‐related. This suggests that these symptoms should be closely monitored and resolved when experienced by patients. In our study, pharmacists also recorded infrequent AEs with a high likelihood of being medicine‐related. Detecting and monitoring these uncommon AEs can be valuable in preventing medicine‐related harm. For example, pharmacists recorded symptoms such as unsteadiness, unconsciousness, and vertigo at low frequencies (< 1%), but with a high probability (50%–100%) of being medicine‐related. These symptoms are known risk factors for falls, which are among the most common medicine‐related AEs in aged care facilities [35, 36]. Strategies such as the use of structured symptom checklists, electronic health record alerts, and the integration of decision support systems or trigger tools may support pharmacists in more effectively identifying and documenting these infrequent but serious AEs, thereby reducing underreporting and enhancing patient safety in aged care facilities [16, 40].

Detecting and reporting medicine‐related AEs are important for ensuring patient safety in the healthcare system [41]. In our study, pharmacists recorded a notable number and variety of AEs; however, underreporting and discrepancies were still evident compared to the AMEs detected by the clinical panel. Despite a statistically significant association, the comparison between pharmacists' records and the panel showed that only 14% of AMEs identified by the panel were documented in the pharmacists' notes, with a low level of agreement (*κ* = 0.074). These findings indicate notable discrepancies in AME recording as well as many AEs experienced by residents went unrecorded by pharmacists. Underreporting and inconsistencies in documenting AMEs may be attributed to under‐detection of these events and variability in clinical judgment [42]. In healthcare systems, underreporting of such events by healthcare professionals, including pharmacists, remains a common issue due to various contributing factors [43]. The underrecording of AMEs in pharmacists' notes observed in our study may be due to pharmacists' unfamiliarity with writing progress notes, as ReMInDAR was a new service [29, 30], and many of the participating pharmacists had no prior experience with writing progress notes. This may mean that pharmacists did not record all they observed. The second half of the trial also occurred during the COVID pandemic which may have affected pharmacists' ability to attend the facility and record. Underreporting of AMEs by pharmacists in our study may also be related to the methods and resources pharmacists used to identify AMEs [44]. In our study, pharmacists recorded AEs by reviewing residents' care records and conducting interviews. A previous study highlighted that only 22.0% of the AEs or symptoms were documented in medical care records [45].

The underreporting or recording of AMEs by pharmacists in our study underscores the need for targeted training and the development of strategies to improve the documentation and reporting of medicine‐related AEs. The use of a standardized and validated tool to track changes in signs and symptoms could be a potential strategy to support pharmacists in systematically identifying a range of medicine‐related AEs in routine practice [26, 27, 46, 47, 48]. Evidence suggests that symptoms reported by patients using validated structured symptoms checklists are important in identifying symptomatic AMEs experienced by patients [28, 49, 50, 51]. Moreover, the integration of digital technologies into pharmacy practice may provide additional opportunities to enhance the early identification and reporting of medicine‐related AEs [52, 53]. Digital technologies such as structured medication review templates, electronic health records with embedded clinical alerts and clinical decision support systems may support pharmacists in improving the consistency in recording and reporting medicine‐related AEs [54, 55, 56].

In our study, the qualitative comparison between frequently recorded medicine‐related symptoms by pharmacists and items in established assessment tool (PHASE‐20) demonstrated substantial overlap, suggesting that this tool effectively capture many of the symptoms pharmacists commonly observe in practice. This alignment suggests that using this tool may support pharmacists in identifying AMEs, which may help reduce underreporting of AMEs in routine pharmacy practice. Pharmacists should take into account symptoms reported by patients, as they may be indicative of AMEs [20, 39]. Studies showed that 53%–72% of the symptoms reported by patients were related to their medications in use [21, 26]. Seeking and routinely monitoring symptoms reported by patients using validated tools, such as predefined and structured medicine‐related symptom checklists, may provide additional information for pharmacists to identify AMEs [20, 28]. Medicine‐related symptom checklists such as PHASE‐20 and PROMISE tools may enhance awareness and recall of such symptoms among patients [26, 27, 28], thereby encouraging and guiding them to report potential medicine‐related AEs to pharmacists [28, 50].

### 4.1 Limitations of the Study

This study was a secondary analysis of data from the ReMInDAR trial and therefore shares its inherent strengths and limitations [29]. As the original trial was conducted in controlled aged care facilities, the findings may not be generalizable to all routine practice. As a secondary study, the sample size was not specifically powered to detect AMEs as a primary outcome. To address this limitation, we compared pharmacist‐recorded AMEs with those identified by the clinical panel from the same population with equal sample size. We also conducted a comprehensive review of all pharmacists' progress notes (575 notes and 309 recommendations) recorded during the provision of pharmacist‐led services for 115 residents. A preformatted data extraction form was used to extract AE information and corresponding dates, and the extracted data were subsequently reviewed by a second pharmacist to minimize the risk of missing any AEs during the initial extraction from the progress notes. Another limitation is the potential for documentation bias, as variability in how different pharmacists recorded progress notes may have affected the consistency and completeness of the data. Despite these limitations, the study provides valuable insights into the types and prevalence of AMEs, and symptoms recorded by pharmacists in aged care facilities. These findings may help prioritize specific events for monitoring during pharmacist‐led services. The findings also highlight gaps in the recording of AMEs and emphasize the need for strategies to improve pharmacists' identification of medicine‐related AEs. Future studies specifically designed and adequately powered to evaluate AMEs recorded by pharmacists during routine practice are warranted.

## 5 Conclusion

Our study found that the majority of symptoms recorded by pharmacists for residents in aged care facilities are medicine‐related; however, underreporting was observed. Future research should focus on exploring the root causes of low recording of AMEs by pharmacists as well as developing strategies for assisting pharmacists to identify and monitor AMEs. Considering the symptoms listed in the PHASE‐20 medicine‐related symptom assessment tool matched with the majority of events recorded by pharmacists, further research is warranted to determine if this tool can assist pharmacists in improving the detection and monitoring of AMEs in routine practice.

## Author Contributions

**Abebe Basazn Mekuria:** study design, data analysis, interpretation, drafting and editing the manuscript. **Elizabeth E. Roughead:** conceptualization, study design, interpretation, manuscript review and editing, and supervision. **Andre Q. Andrade:** study design, interpretation, manuscript review and editing, and supervision. **Renly Lim:** study design, interpretation, manuscript review and editing, and supervision. **Debra Rowett:** study design, interpretation, manuscript review and editing, and supervision.

## Disclosure

This manuscript, or any part of it, has not been previously published or presented at any conference prior to this submission.

## Ethics Statement

The ethics approval for the trial has been obtained from both the Human Research Ethics Committee at the University of South Australia (ID: 0000036440) and the University of Tasmania (ID: H0017022).

## Conflicts of Interest

The authors declare no conflicts of interest.

## Supporting information


**Data S1:** pds70261‐sup‐0001‐Supinfo1.docx.

## Data Availability

The datasets generated by the current study are available from the data custodian upon request.
