# Supplementary material for: Detection of Adverse Medicine Events by Pharmacists in Residential Aged Care Facilities: Secondary Analysis of Data From ReMInDAR Trial
Source: Pharmacoepidemiol Drug Saf. 2025 Nov 6;34(11):e70261. doi: 10.1002/pds.70261 (PMC12592833; doi:10.1002/pds.70261)
Supplement: Supplementary file 1 — Data S1: pds70261‐sup‐0001‐Supinfo1.docx. [file PDS-34-e70261-s001.docx]

**Supplementary 1**: The qualitative comparison between the most prevalent medicine-related symptoms recorded by pharmacists and symptoms listed in medicine-related symptoms assessment tools.

| **Frequently detected medicine-related symptoms (frequency ≥ 1.0% and medicine-likeliness ratio of ≥ 40.0%)** | **Matched tools’ items with the most prevalent medicine-related symptoms recorded by pharmacists** | |
| --- | --- | --- |
|  | **PROMISE** | **PHASE-20** |
| Fall |  | Dizzy/unsteady/high risk of falls |
| Swelling* |  | Swollen legs/ankles |
| Constipation | Constipation | Constipation |
| Nocturia/Incontinence |  | Frequent urination/incontinent of urine |
| Shortness of breath |  | Short of breath |
| Bleeding | Bruises, bleedings |  |
| Dizziness | Dizziness, vertigo, fainting | Dizzy/unsteady/high risk of falls |
| Drowsiness | Drowsiness |  |
| Nausea/vomiting | Nausea, vomiting | Nausea/vomiting |
| Cognitive impairment |  | Forgetful |
| Tiredness | Weakness, tiredness | Tired/exhausted |
| Bruising | Bruises, bleedings |  |
| Rash |  | Itching/rash |
| Confusion |  |  |
| Diarrhoea | Diarrhoea | Diarrhoea |
| Dry mouth | Dry mouth/ thirst, mouth complaints | Dry mouth |

* It includes swelling of the ankles, legs, and arms; PHASE-20 = PHArmacotherapeutical Symptom Evaluation, 20 questions; PROMISE = Patient Reported Outcome Measure, Inquiry into Side Effects.
